# Operation Notes Illustrated With Digital Images

**DOI:** 10.1080/13577140500094362

**Published:** 2005

**Authors:** R. M. Haywood, M. Heaton, T. A. McCulloch, M. Sokal, A. G. B. Perks

**Affiliations:** 1 Department of Plastic Reconstructive and Burns Surgery Nottingham City Hospital Hucknall Road Nottingham NG5 1PB UK; 2 Department of Histopathology Nottingham City Hospital Hucknall Road Nottingham NG5 1PB UK; 3 Department of Oncology Nottingham City Hospital Hucknall Road Nottingham NG5 1PB UK

## Abstract

We would like to report on our experience of illustrating our operation notes with pre-, per- and post-operative digital images.


*Sarcoma*, March/June 2005; 9(1/2): 21–24

Taylor & Francis

Taylor & Francis Group

**ORIGINAL ARTICLE**

### Operation notes illustrated with digital images

R. M. HAYWOOD[1], M. HEATON[1], T. A. McCULLOCH[2], M. SOKAL[3], & A. G. B. PERKS[1]

[1]*Department of Plastic, Reconstructive and Burns Surgery,* [2]*Department of Histopathology, and*
[3]*Department of Oncology, Nottingham City Hospital, Hucknall Road, Nottingham NG5 1PB, UK*

**Abstract**
We would like to report on our experience of illustrating our operation notes with pre-, per- and post-operative digital images.

**Keywords:** *Operation note, digital image*

### Method

Templates for several common types of operations performed by the authors were created in Microsoft Word 2000 (Figure 1). The essential elements of these templates are: patient name, number and date of birth, date of operation, operation title, surgeon and assistants, anaesthetic, incision, findings, closure and post-operative instructions.

Pre-operatively patients are asked to give written consent for digital photographs to be taken pre-, per- and post-operatively to be used to generate an illustrated operation note and for the photographs to be used for teaching, research and publications.

Pre-, per- and post-operative photographs are taken with a digital camera and transferred to a laptop computer via a PCMIA memory card adaptor.

A new operation note is created with an appropriate template and the patient and operative details typed. The images are inserted using the 'insert' menu selecting 'picture' and 'from file'. Once the image is inserted it is reduced to an appropriate size. To enable easier movement of the image around the operation note the layout of the picture is changed by 'double clicking' the picture to highlight the 'format' menu, then selecting 'layout' and then selecting the 'tight' wrapping style. The image can be labelled using the 'callout' option within 'autoshapes'. The operation note is printed using a colour printer in theatre, signed, stapled to the anaesthetic sheet and consent form and then placed in the patient's notes (Figure 2). A copy of the operation note, including the digital images is attached to the pathology request form. The operation note is saved on the surgeon's laptop computer along with the original digital images. These are filed in folders according to the diagnosis and individually using a combination of the patient's name and hospital number.

Recent refinements to this system have involved the establishment of a server computer within the plastic surgery department to hold all the digitised images. This computer is linked via the hospital network to a terminal within the medical illustration department to facilitate the transfer and storage of all of images taken both by clinicians and medical photographers. This image store acts as a convenient central location for audit, research and educational purposes. Ultimately it is envisaged that this information would be available to members of the department whilst attending peripheral hospitals and clinics via remote access software, using a system of encryption and digital certificates to ensure availability only to suitably authorized persons. It is hoped that we can add the operation notes to this server as well.

### Discussion

Digital images have been used by previous authors to illustrate an operation note [1,2]. Their technique

Correspondence: Mr R.M. Haywood, Department of Plastic, Reconstructive and Burns Surgery, Nottingham City Hospital, Hucknall Road, Nottingham NG5 1PB, UK. Tel: 44 115 969 1169, ext 46428. E-mail: richardhaywood@ntlworld.com

ISSN 1357–714X print/ISSN 1369–1643 online © 2005 Taylor & Francis Group Ltd
DOI: 10.1080/13577140500094362

**OPERATION NOTES**
DEPARTMENT OF PLASTIC, RECONSTRUCTIVE AND BURNS SURGERY
NOTTINGHAM CITY HOSPITAL

**Name:**
**Number:**
**Date of Birth:**

**Date:**
**Consultant:**

**Title:**

**Surgeon:** Mr R M Haywood
**Assistant:** Mr
**Anaesthetic:** LA GA

**Incision:**

**Findings:**

**Procedure:**

**Closure:** deep dermal
skin

**Post op:** ROS days
OPC weeks

**Signed :** Mr R M Haywood FRCS

Figure 1. Blank word template.

stored the images on disk in the patients' notes or a computer and therefore required a laptop or personal computer to view these images. Our technique allows the images to be readily seen in the patients' notes and copies sent to peripheral clinics and other specialists of the multidisciplinary team to aid further management. The obvious advantage of an operation note illustrated with photographs is the extra information that can be portrayed. Doctors, nursing staff and allied ancillary medical staff have found these operation notes particularly useful in the post-operative care of the patient. Knowing exactly what to expect under a dressing allowed easier planning of dressing changes. Early identification of any complications is made easier by direct comparison of the patient and the image on the operation note. The presence of the illustrated operation note, with the appropriate labelling, also facilitates far greater ease of handling and orientation of the specimen within the histopathology department. Scrupulous attention to the margins is essential in the reporting of soft tissue sarcomas. Reference to the illustrations in the operation note allows precise identification of different margins and places the specimen within an anatomical context. Clear categorisation of margin status is essential if further excisions are to be contemplated and in the planning of adjuvant therapy, such as radiotherapy. Thus in the post-operative follow-up clinic these illustrated operation notes allow accurate identification of incompletely excised tumour borders when allied with the histology report, allow easier planning and

### OPERATION NOTES

DEPARTMENT OF PLASTIC, RECONSTRUCTIVE AND BURNS SURGERY
NOTTINGHAM CITY HOSPITAL

**Name:** XXXXXXXXX
**Number:** XXXXXX
**Date of Birth:** XXXXX

**Date:** 12/9/2001
**Consultant:** Mr Perks

**Title:** Excision sarcoma left scapula reconstruction with parascapular flap

**Surgeon:** Mr Perks, Mr R M Haywood
**Assistant :** Mr Misra, Dr De Kerpal

**Anaesthetic:** GA

**Incision:** 20 mm margin marked

**Findings:** Deep margin macroscopically clear of tumour, small incision into abnormal tissue marked with suture

**Procedure:** Tumour excised with periosteum and part of trapezius
Bipolar haemostasis

Parascapular flap raised on descending branch of circumflex scapular vessels

**Closure:** 3/0 monocryl deep dermal
3/0 monocryl skin

**Post op:** Drain out < 40 ml in 24 hours
OPC 6 weeks in sarcoma clinic

**Signed:** Mr R M Haywood FRCS

Marker suture

Flap Pedicle

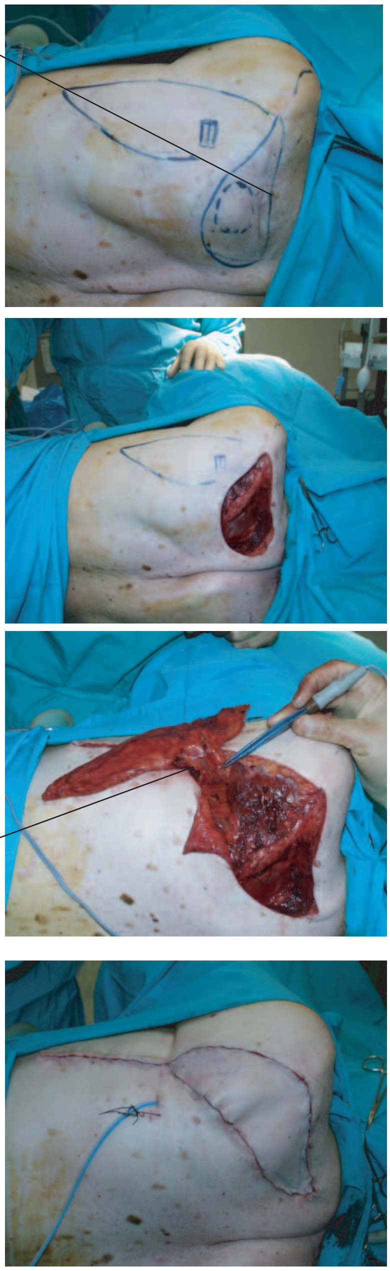

Figure 2. Completed operation note.

discussion of further surgery or treatment with the patient. The illustrations give the radiotherapist a very accurate picture of the tumour bed to allow easier planning of radiotherapy fields. If the patient develops keloid or hypertrophic scarring, the original zone of surgical trauma is accurately demonstrated. If stored on a secure laptop they can prove invaluable when a copy of the original note has not reached the peripheral hospital clinic before the patients follow-up appointment.

Our templates act as aide memoirs with the surgeon prompted to fill in essential information by headings, this has been shown to improve the quality of information and reduction of abbreviations in an operation note [3]. We have found that with several different tailored templates completing these operation notes takes no longer than writing or drawing the operation note by hand. The information is clear, legible and immediately available, we feel this idea could be utilised in many different surgical specialities. Storage at a central location would allow access of images to all suitably authorized members of the department for the purposes of audit, teaching and education. Access to this store from peripheral hospitals would facilitate consultations and reduce the flow of paper between these locations. Adequate mechanisms are now available to ensure secure storage of and access to digital images.

### References

[1] Senior MA, Southern SJ, Nishikawa H, Chumas PD. The digital op note. British Journal of Plastic Surgery 2000;53(7):633.

[2] Rhodes ND, Southern SJ. Digital operation notes: A useful addition to the written record. Annals of Plastic Surgery 2002;48(6): 571–573.

[3] Bateman ND, Carney AS, Gibben KP. An audit of the quality of operation notes in an otolarygology unit. Journal of the Royal College of Surgeons of Edinburgh 1999;44: 94–95.

